# New York Letter

**Published:** 1879-06

**Authors:** 


### Domestic Correspondence.

ARTICLE XVI.

NEW YORK CITY, May 1879.

*Messrs. Editors:*—The question of what to do with our tenement-house population is becoming a serious one, both to sanitarians and economists. The five hundred thousand people who live here in these structures contribute to more than two-thirds of the city's mortality and a still larger per cent. of its crime. It would seem as though decency, morality and health diminished as the overcrowding of houses and homes increased. And not much can be expected when four families occupy a single room and the domestic life of each is limited by a chalk line. We have had a "Tenement-house Sunday" when the matter was discussed in all the pulpits, and this has been followed by a number of public meetings and the final organization of a company for building model tenement houses. The great difficulty lies in the small size of Manhattan Island. For no matter how model a tenement may be, it must be a many-storied one, and such can never be so good or effective as a number of small houses or cottages like those Philadelphia gives to her workmen. We are attempting these, however, also, and it is believed that they will be a financial success in spite of high rents. In addition to this, large model tenements are projected and some already built. The principal features of these are that each floor is separate and reached only by outside staircases, and that careful provision is made for light, air and the removal of garbage.

The County Medical Society has been in a state of mind recently over the abuses of medical charity. We have six large dispensaries in the city, and from thirty to forty smaller and more or less special ones. These treat gratuitously about a fourth of the city's population, that is, from 250,000 to 300,000, in the course of the year. Of course a great many of these can well afford to pay the doctor and the younger and struggling members of the profession are thus directly injured. Yet the doctors are chiefly to blame for this after all. It is they who have encouraged the unnecessary multiplication of dispensaries; there is a constant struggling and wire-pulling for appointments among them, and a constant effort to increase the number of the patients in order to have an interesting service. While such things exist, while there are plenty of doctors ready to submit to any rules, however humiliating, for the sake of an appointment, it is idle to expect any radical reform. Indeed the trouble lies deeper than this. The profession is too over-crowded for healthy competition and the schools are grinding out more and more every year. We have nearly twenty-eight hundred physicians of one kind and another in the city, and besides the fact of superfluity, not more than a quarter are members of societies that could enforce discipline. We presume therefore that the county society after heating itself with a few evenings of debate will appoint a committee and the committee will report, and resolutions will be adopted and things will go on much as before.

We by no means wish, however, to give the opinion that things are in a desperately gloomy state. As far as appearances indicate the physician continues to earn his living and to live in harmony with his neighbors and brethren. And though a quarter of a million get their pills and paregoric free at the dispensaries, a few are left to call at the office on the usual terms. Besides it is probable that some amelioration at least of the present abuses can be obtained. There is a rich hospital here, the New York Hospital, which at the instigation of a homœopathic trustee, has offered publicly medical advice to any one on condition of the receipt of a modest monthly stipend, amounting to one dollar. This is a disgraceful prostitution of medical service, which it does not seem possible can continue in the face of the strong sentiment against it among all the members of the profession except those who are bribed to acquiesence by their hospital appointment. The plan of obliging every patient who can do so to pay a small sum for medicine and prescription has been introduced quite generally into the dispensaries and is considered a success by most of the officers of those institutions. In London, where evils much like our own exist, the Provident Dispensary system is being tried to some extent. This consists in forming persons whose wages do not exceed $6 or $8 a week into a sort of Health Insurance Company. A small sum is paid every week into the general fund and in return the medical officers take care of the members when sick. Such societies are liable to abuses and all have not succeeded, but when carefully conducted they may be very efficient substitutes for or auxiliaries of the ordinary dispensary.

One of the wealthiest and best conducted of the city's hospitals is the Roosevelt. It has a magnificent endowment, is thoroughly built and perfectly managed by its superintendent, Dr. Paine. It is constructed to a certain extent on the pavilion plan, the wards occupying two long separate buildings, one for the medical and the other for surgical cases. The buildings are connected only by corridors. The antiseptic treatment was early introduced here and carried out in every detail by Dr. Weir, who is one of the very few surgeons if not the only one who does seem always to be mindful of the minutiæ. The results have been satisfactory here as in the other city hospitals. Not all the visiting surgeons use it uniformly, but when anything of especial importance is to be done it is noticeable that they consider it best to give the patient whatever additional chance it may furnish. Though abandoned for a time at the Woman's Hospital as I wrote in my last letter, it has now been resumed there. At Roosevelt it is now succeeding admirably in compound fractures. At first it was found that though the limb did pretty well, yet necrosis was very apt to occur. The plan was then adopted of first opening the wound, cleaning it thoroughly and sawing off any ragged ends of bones, the dressing being applied after this as usual. Since then necrosis has not occurred.

A remarkable illustration of what a Lister dressing may do occurred a short time ago. A boy was brought in with a severe lacerated wound of the scalp, extending transversely across the vertex, tearing up much of the scalp and nearly removing the left ear which hung only by a little skin and subcutaneous tissue. The wound was dressed antiseptically and in two days it had all healed except of course the cartilaginous part of the ear which, however, subsequently gave no trouble. Instead of the carbolized gauze usually employed by Lister, jute is used, which has been soaked in a solution of benzine and carbolic acid. It is a little cheaper and more efficient than the gauze.

The gum bandage is being used very extensively; in synovitis, arthritis, in sprains and bruises it seems to act remarkably well. Its magical effect on old ulcers is not so prominent. The ordinary treatment for these is to first wash them off with carbolized water, 1 to 20, then cover them with cotton that has been soaked in a saturated solution of boracic acid; over this the gum bandage is applied. Under this treatment the ulcers soon fill up with granulations, but cicatrization is by no means rapid, and it seems to be thought by the staff that those who have secured such wonderful results with this bandage have mistaken granulation for cicatrization. Buck's extension is still the popular method here of treating fractures of the thigh, although at some of the other hospitals the double-inclined plane may be seen.

Roosevelt is one of the hospitals which seem to ignore the fact that such institutions are built not to cure the sick, but to furnish clinical material for professors and students. In spite of its excellent run of cases very little teaching is done over them, and students are rarely seen in the wards. Even a proposal of a New York pathologist to build a pathological laboratory there at his own expense, for study and teaching, was refused. The contributions of the hospital therefore to medical and surgical science come through its attending medical officers. It is well enough perhaps that there should be some hospitals that are almost purely humanitarian institutions.

